# Author Correction: Indirect tail states formation by thermal-induced polar fluctuations in halide perovskites

**DOI:** 10.1038/s41467-019-09172-3

**Published:** 2019-03-05

**Authors:** Bo Wu, Haifeng Yuan, Qiang Xu, Julian A. Steele, David Giovanni, Pascal Puech, Jianhui Fu, Yan Fong Ng, Nur Fadilah Jamaludin, Ankur Solanki, Subodh Mhaisalkar, Nripan Mathews, Maarten B. J. Roeffaers, Michael Grätzel, Johan Hofkens, Tze Chien Sum

**Affiliations:** 1 Institute of Electronic Paper Displays, South China Academy of Advanced Optoelectronics, South China Normal University, Guangzhou, Guangdong Province 510006 China; 2 Division of Physics and Applied Physics, School of Physical and Mathematical Sciences, Nanyang Technological University, 21 Nanyang Link, Singapore, 637371 Singapore; 3 Department of Chemistry, KU Leuven, Celestijnenlaan 200F, B-3001 Leuven, Belgium; 4 Centre for Surface Chemistry and Catalysis, KU Leuven, Celestijnenlaan 200F, 3001 Leuven, Belgium; 5 CEMES/CNRS, University of Toulouse, 31055 Toulouse, France; 6 School of Materials Science and Engineering, Nanyang Technological University, 50 Nanyang Avenue, Singapore, 639798 Singapore; 7 Energy Research Institute @NTU (ERI@N), Research Techno Plaza, X-Frontier Block Level 5, 50 Nanyang Drive, Singapore, 637553 Singapore; 8 Laboratory of Photonics and Interfaces, Department of Chemistry and Chemical Engineering, Swiss Federal Institute of Technology, Station 6, 1015 Lausanne, Switzerland

Correction to: *Nature Communications*; 10.1038/s41467-019-08326-7; published online 29 January 2019.

The original version of this article incorrectly listed the present address of Bo Wu as ‘Present address: Institute of Electronic Paper Displays, South China Academy of Advanced Optoelectronics, South China Normal University, Guangzhou, Guangdong Province 510006, China’. This is the author’s primary affiliation. This has been corrected in both the PDF and HTML versions of the article.

