# Supplementary material for: Indirect tail states formation by thermal-induced polar fluctuations in halide perovskites
Source: Nat Commun. 2019 Jan 29;10:484. doi: 10.1038/s41467-019-08326-7 (PMC6351600; doi:10.1038/s41467-019-08326-7)
Supplement: Supplementary file 1 — Supplementary Information [file 41467_2019_8326_MOESM1_ESM.docx]

Supplementary Information for

**Indirect Tail States Formation by Thermal-induced**

**Polar Fluctuations in Halide Perovskites**

Bo Wu^1,2#^, Haifeng Yuan^3#^, Qiang Xu^2^, Julian A Steele^4^, David Giovanni^2^, Pascal Puech^5^, Jianhui Fu^2^, Yan Fong Ng^6^, Nur Fadilah Bte Jamaludin^6^, Ankur Solanki^2^, Subodh Mhaisalkar^6,7^, Nripan Mathews^6,7^, Maarten B. J. Roeffaers^4^, Michael Grätzel^7,8^, Johan Hofkens^3^*, & Tze Chien Sum^2^*

^1^Present Address: Institute of Electronic Paper Displays, South China Academy of Advanced Optoelectronics, South China Normal University, Guangzhou, Guangdong Province, 510006, China

^2^Division of Physics and Applied Physics, School of Physical and Mathematical Sciences, Nanyang Technological University, 21 Nanyang Link, Singapore 637371

^3^Department of Chemistry, KU Leuven, Celestijnenlaan 200F, B-3001 Leuven, Belgium

^4^Centre for Surface Chemistry and Catalysis, KU Leuven, Celestijnenlaan 200F, Leuven, 3001, Belgium

^5^CEMES/CNRS, University of Toulouse, 31055, Toulouse, France

^6^School of Materials Science and Engineering, Nanyang Technological University, Nanyang Avenue, Singapore 639798

^7^Energy Research Institute @NTU (ERI@N) Research Techno Plaza, X-Frontier Block Level 5, 50 Nanyang Drive, Singapore 637553

^8^Laboratory of Photonics and Interfaces, Department of Chemistry and Chemical Engineering, Swiss Federal Institute of Technology, Station 6, Lausanne 1015, Switzerland

Correspondence and requests for materials should be addressed to T. C. S. (email: [Tzechien@ntu.edu.sg](mailto:Tzechien@ntu.edu.sg)) or J. H. (email: [johan.hofkens@chem.kuleuven.be](mailto:johan.hofkens@chem.kuleuven.be)).



**Supplementary Figure 1. Images and XRD patterns of perovskite single crystals.** (a) CsPbBr_3_ (b) FAPbBr_3_ and (c) MAPbBr_3_. The corresponding XRD patterns are shown in (d)-(f).





**Supplementary Figure 2. Absorbance of perovskite single crystals measured using diffuse reflectance spectroscopy.** Temperature-dependent absorbance of (a) CsPbBr_3_ (b) FAPbBr_3_ and (c) MAPbBr_3_ SCs. The original experimental data were converted to absorbance by the Kubelka-Munk formula: *k*/*s* = (1-*r*)2/2*r*, where *k* and *s* are absorption and scattering coefficients, *r* is the diffuse reflectance. Considering that the scattering varies slowly over a small frequency region, we can therefore use *k*/*s* to represent the absorbance profile.^1^





**Supplementary Figure 3.** **The temperature-dependent Peak 2 fitted with the Varshni equation**: $E_{\text{g}}=E_{0}-\alpha T^{2}/(T+\beta)$, where *α* and *β* are fitting parameters that are characteristic of a given semiconductor. *E*_0_ is the band gap at 0 K. We obtained *E*_0_=2.32 ± 0.01 eV, α=4.9 ± 0.5 × 10^-4^ eV/K, β = 190 ± 60 K for CsPbBr_3_ between 77 K and 300 K. We did not perform the same fitting for the other two perovskites, since there are phase transitions in this temperature range which changes the band gap abruptly.

**Supplementary Figure 4. SEM image and PL kinetics of MAPbBr_3_ film prepared with varied precursor concentration.** (a) Representative SEM images of MAPbBr_3_ films with different precursor concentration. (b) The PL decay kinetics of the MAPbBr_3_ films prepared with different precursor concentration. Higher precursor concentration yields longer carrier lifetimes approaching those of single crystals. (c) Excitation density-dependent PL intensity (400 nm excitation). (d) Normalized PL profiles of MAPbBr_3_ film (1M) with varied excitation density.

**Supplementary Figure 5. AFM images MAPbBr_3_ films prepared with varied precursor concentration.** (a) 0.25 M, (b) 0.5 M, (c) 0.75 M and (d) 1 M. The RMS are 4.8 nm, 4.8 nm, 7.7 nm and 6.5 nm, respectively.





**Supplementary Figure 6. Time-resolved photoluminescence of MAPbBr_3_ single crystals with 400 nm excitation.** (a) Pseudo-color map of MAPbBr_3_ SC emission with time (*x*-axis) and energy (wavelength, *y*-axis). (b) Selected time slices of MAPbBr_3_ emission spectra (dots) and fitting results using non-negative matrix factorization (NNMF) deconvolution method. The (c) spectra and (d) dynamics of the two extracted emission species after deconvolution. Peak 1 possess a short effective lifetime *τ*_1_ ~ 15 ns (*i.e.*, the time for the PL intensity to drop to 1/*e* of its initial value), while Peak 2 has a much longer effective lifetime *τ*_2_ ~ 170 ns. Peak 1 mainly originates from the surface part while Peak 2 is an integration from the surface to deep bulk. The lifetime difference arises from surface recombination and carrier diffusion reported previously.^1^





**Supplementary Figure 7.** A comparison of MAPbBr_3_ SC PL detected at (a) left *σ*_+_ and (b) right *σ*- circular polarizations. The excitation wavelength is 473 nm. Right circularly polarized PL for CsPbBr_3_ SC upon (c) 532 nm (d) 473 nm and (e) 400 nm optical excitation with right *σ*- (blue) or left *σ*_+_ (red) circularly polarized laser pulses.





**Supplementary Figure 8. PL profiles of lead bromide perovskite SCs at low temperature (45 K).** Peak 1 and 2 can be easily discerned. Peak 3 is also observed in MAPbBr_3_ SC, which is most likely the phonon replica of Peak 1.





**Supplementary Figure 9. Temperature-dependent FWHMs of (a) CsPbBr_3_ SC and (b) MAPbBr_3_ TF.** The broadening mechanisms of both peaks can be explained by the Fröhlich coupling of Pb-Br LO phonons.

**Supplementary Figure 10**. **Examples of fitting the low-energy portion of the Raman spectrum recorded from CsPbBr_3_ at (a) 300 K and (b) 80 K.** The two differing components used to fit the experimental data have been deconvolved, with their relative contributions explicated. For the spectrum recorded at the lower temperature of 80 K, the band near 75 cm^-1^ exhibited moderate splitting, complicating the fit and the use of a single Lorentz oscillator to account for its contribution. As such, for spectra experiencing relatively large splitting of this band (*i.e.*, for temperatures 160 K and below), 2 Lorentz terms are used to fit this band rather than a heavily overdamped single line-shape which would otherwise account for this spectral feature.

**Supplementary Figure 11. Examples of the calculated band structures of CsPbBr_3_ SC at zero and elevated temperatures.** (a) The simulated band structure of CsPbBr_3_ at zero temperature. (b) The simulated band structure of CsPbBr_3_ using the typical distorted crystal structure as shown in the main text at 300 K. Right: zoom-in band structure in Γ-Y direction. Figure (b) does not represent the accurate structure of CsPbBr_3_ at 300 K. It is a typical instantaneous structure and the crystal band structure is dynamically changing, resulting in dynamical indirect band edge.





**Supplementary Figure 12. Recombination kinetics in lead bromide perovskites.** (a) Typical PL kinetics of FAPbBr_3_ SC at low temperature (110 K) showing high intensity-dependence with 800 nm 2PA excitation. (b) Temperature-dependent *k*_1_ for different SCs and PCs. The details of the *k*_1_ and *k*_2_ extraction for SCs are given in Supplementary Note 3.

**Supplementary Figure 13. Normalized PL spectra versus Incident excitation density for all the lead bromide single crystals.** No amplified spontaneous emission (ASE) is observed even when the carrier concentration reaches 10^20^ cm^-3^.





**Supplementary Figure 14. PL properties of CdS SC excited with 400 nm at room temperature.** (a) PL profiles of CdS SC with different excitation density. (b) Linear dependence of PL intensity on excitation density. (c) The PL lifetime with excitation density. The PL signatures indicate that the CdS emission is dominated by excitonic states.





**Supplementary Figure 15. PL intensity vs. Excitation fluence with 800 nm excitation for MAPbBr_3_ SC at 77 K.** *λ*= 4.5 was extracted using a power-law fitting: *I* ∝ *n^λ^*, where *n* is the excitation density. This indicates a quadratic dependence of PL on carrier concentration (*λ*= 2.2), *i.e.*, the emission in bulk part of the crystal at 77 K is still dominated by free carriers.

**Supplementary Tables**

**Supplementary Table 1. Recombination coefficients of lead halide perovskites from the literature.**

| **Sample** | ***k*_1_ (s^-1^)** | ***k*_2_ (cm^3^ s^-1^)** | **Technique** | **Morphology** | **Reference** |
| --- | --- | --- | --- | --- | --- |
| MAPbBr_3_ | 2.70E6 | 8.00E-11 | Steady-state photoconductivity | Single crystal | *Nat. Comm.* **7**:12253 (2016) ^2^ |
| MAPbI_3_ | 2.00E7 | 1.85E-8 | TCSPC | Thin Film | *Energy Environ. Sci.*, **10**, 509 (2017)^3^ |
| MAPbI_3_ | 6.67E7 | 2.00E-9 | TRPL | Thin Film | *Nat.Mater.*,**16**,115-120, (2017) ^4^ |
| MAPbI_3-x_Cl_x_ | 1.20E7 | 1.10E-10 | THz photoconductivity | Thin Film | *Energy Environ. Sci.*, **7**,2269 (2014) ^5^ |
| FAPbI_3_ | - | 1.00E-10 | THz photoconductivity | Thin Film | *Adv. Mater.*, **27**, 7938–7944 (2015) ^6^ |
| FAPbBr_3_ | - | 1.00E-9 | THz photoconductivity | Thin Film | *Adv. Mater.*, **27**, 7938–7944 (2015)^6^ |
| MAPbI_3_ | 4.00E7 | 0.5 to 5.3E-9 | TCSPC | Thin Film | *Adv. Funct. Mater.* **26**, 4283–4292 (2016) ^7^ |
| MAPbI_3_ | - | 1.10E-9 | TA | Thin Film | *J. Mater. Chem. A*, **3**,9285 (2015)^8^ |
| MAPbI_3_ | 1.49E7 | 6.10E-11 | THz photoconductivity | Thin Film | *Adv. Funct. Mater.*, **25**, 6218–6227 (2015)^9^ |
| MAPbI_3_ | - | 3.70E-9 | TRPL | Small crystal (<100 nm) | *Acc. Chem. Res.*, **49**, 536−544 (2016) ^10^ |
| MAPbI_3_ | - | 6.20E-10 | TRPL | Large crystal (>1 μm) | *Acc. Chem. Res.*, **49**, 536−544 (2016) ^10^ |
| MAPbBr_3_ | 2.70E7 | 4.90E-10 | TA | Thin Film | *J. Phys. Chem. Lett.*, **6**, 4688−4692, (2015) ^11^ |
| MAPbI_3_ | 7.69E7 | 1.50E-10 | TA | Thin Film | *J. Phys. Chem. Lett.*, **6**, 4688−4692, (2015)^11^ |
| MAPbI_3_ | 1.79E7 | 1.70E-10 | TRPL | Thin Film | *J. Am. Chem. Soc.*, **136**, 11610−11613 (2014)^12^ |
| MAPbI_3_ | - | 2.30E-9 | TA | Thin Film | *Nat. Photonics*, **8**, 737 (2014)^13^ |
| MAPbI_3_ | 5E6 to 1.5E7 | 0.87 to  9.4E-10 | THz photoconductivity | Thin Film | *Adv. Mater.*, **26**, 1584–1589 (2014)^14^ |
| MAPbI_3_ | 3.2E6 | 1.30E-10 | TRPL | Thin Film | *Phys. Rev. Appl.*, **2**, 034007 (2014) ^15^ |

**Supplementary Notes**

**Supplementary Note 1. Origins of the dual PL peaks in lead halide perovskite SCs**

Below, we provide a succint review of the various viewpoints and show that these existing postulates are inadequate in light of our latest results.

(1) *Lateral inhomogeneities and coexistence of structural phases*. This interpretation attributed the two PL peaks to originate from the inhomogeneous distribution of particle size or the coexistence of small crystallites of room-temperature phase with the low-temperature orthorhombic phase.^16-18^. Although inhomogeneity is valid for crystallites with varied size, this assignment does not hold for larger mono-crystalline crystals. Despite the absence of any phase transition over the range 77 K – 300 K, CsPbBr_3_ SCs also exhibit two PL peaks. Furthermore, in all the perovskite phases (cubic, tetragonal and orthorhombic), the thermal dilation effect dominates, leading to an increase of the bandgap with temperature. However, for the Peak 2, we observed a redshift of its position with temperature, which cannot be explained by the existence of another phase crystallite.

(2) *Defect bound carriers or bound exciton emission*. Emission from defect bound carriers or from bound excitons have also been proposed as a possible origin for Peak 2.^19^ Excess PbBr_2_ that was detected in MAPbBr_3_ particles was suspected to introduce shallow radiative defect states. However, neither free-to-bound emission (free *e*/*h* with neutral acceptor/donor) nor bound-exciton emission can explain the quadratic dependence on the excitation power.^20^ Furthermore, Peak 2 is usually prominent in high-quality SCs with long carrier lifetime. In our previous report,^1^ peak 2 is not obvious due to the relatively lower quality of the crystal (~30 ns bulk carrier lifetime). It is unlikely Peak 2 originates from defects or bound exciton emission.

(3) *Surface vs bulk emission*. Previously, we proposed that Peak 1 originates from surface excitons and Peak 2 from bulk excitons due to the difference in lattice strain difference.^1^ This assignment was later supported by O.F. Mohammed’s group,^21,22^ who found that the hydroxyl radicals and hydroxide ions can adsorb on MAPbBr_3_ surface and cause surface strain. However, in light of our new experimental data presented above (*i.e.*, opposite trend for the temperature dependence of Peak 1 and Peak 2 which is highly unusual for surface bandgap vs bulk bandgap), we need to re-examine our earlier stance.

(4) *Free carrier vs exciton emission*. Y-H Qiu *et al* attributed Peak 1 to free carrier emission and Peak 2 to exciton emission after observing that the absorption band edge lies between Peak 1 and Peak 2.^23^ However, in our case, both PL peaks show a quadratic dependence on excitation power. Moreover, the energy difference increases with temperature increase, while exciton binding energies in lead halide perovskites are reported to decrease with increasing temperature. ^24^ Hence, this assignment is deemed to be inadequate.

(5) *Phonon replica*. Peak 2 has also been assigned as the phonon replica of Peak 1, *i.e.*, coupled to one longitudinal optical (LO) phonon of Pb-Br (around 15 meV).^25^ However, we observed a continuous widening of the energy difference between the two peaks with temperature increase. This observation will debunk the phonon replica viewpoint.

(6) *P emission, H emission, and biexciton emission*. Inelastic exciton-exciton scattering (P emission), exciton-electron scattering (H emission) and biexciton emission also exhibit quadratic power dependence.^26^ However, they usually occur in strongly confined systems under low temperatures and high excitation densities.^27^ Moreover, the lifetime for Peak 2 is also too long to be attributed to inelastic exciton scattering as found previously.^27^ The bi-exciton binding energy is usually 0.027-0.3 times that of the Rydberg energy (exciton binding energy).^28^ In the bromide perovskite case, the Rydberg energy is around 30 meV. This means that the bi-exciton binding energy is less than 10 meV, which is much smaller than the difference between Peak 1 and 2.^29^ Hence, these assignments will not satisfactorily account for the origin of peak 2.

(7) *Reabsorption* *effect*. This is the most-frequently assigned origin of the double peaks. The high energy peak (Peak 1) is attributed to emission from the front surface while the low energy peak (Peak 2) is from the back surface following strong attenuation of the high energy region of the emission peak by the reabsorption effect.^30,31^ The position of the low energy peak (Peak 2) in optically-thick crystal is eventually determined by the fundamental absorption edge (*i.e.*, Urbach tail), which originates from thermal and/or disorder induced lattice fluctuations. Thermally-induced lattice fluctuation depends on the phonon occupation number and therefore, it is expected that with increasing temperature, the separation between Peak 1 (high energy peak with no reabsorption) and Peak 2 (low energy peak following reabsorption by the Urbach tail) increases. However, in this work, we found that Peak 2 also appears in thin film samples fabricated with increasing precursor concentration (Figure 2a – main manuscript). Furthermore, our calculations based on uniform carrier distribution involving multi-reflection and multi-reabsorption effects (Supplementary Note 2) shows that reabsorption could only account for less than 10 meV PL energy shift in this case for a thin 220 nm perovskite film. These evidence reinforces one of the main points in our manuscript that the reabsorption effect is inadequate to solely account for the dual peak emissions.

**Supplementary Note 2. Multi-reflection and Reabsorption Model**

The multi-reflection and reabsorption model was adopted from a previous report.^32^ The collected emission light includes:

$\begin{matrix} dI(z,E)=dI_{0}(E)e^{-\alpha(E)z}T_{F}(1+e^{-2\alpha(E)L}R_{\text{B}}R_{\text{F}}+e^{-4\alpha(E)L}R_{\text{B}}^{2}R_{\text{F}}^{2}+...) \\ +dI_{0}(E)e^{-\alpha(E)(2L-z)}T_{F}R_{\text{B}}(1+e^{-2\alpha(E)L}R_{\text{B}}R_{\text{F}}+e^{-4\alpha(E)L}R_{\text{B}}^{2}R_{\text{F}}^{2}+...) \\ =(dI_{0}(E)e^{-\alpha(E)z}T_{F}+dI_{0}(E)e^{-\alpha(E)(2L-z)}T_{F}R_{\text{B}})(1+e^{-2\alpha(E)L}R_{\text{B}}R_{\text{F}}+e^{-4\alpha(E)L}R_{\text{B}}^{2}R_{\text{F}}^{2}+...) \\ =dI_{0}(E)T_{F}\frac{e^{-\alpha(E)z}+e^{^{-\alpha(E)(2L-z)}}R_{\text{B}}}{1-e^{-2\alpha(E)L}R_{\text{B}}R_{\text{F}}} \end{matrix}$ (1)

where d*I*_0_ is the emitted light intensity at the photon energy *E*. *z* is the depth from the front surface. *T*_F_, *R*­_F_, *R*_B_ are the transmittance from the front surface, reflectance from the front surface and reflectance from the back surface respectively. *α*(*E*) is the absorption coefficient at the photon energy *E*, *L* is the crystal thickness, d*I* (*z, E*) is the collected intensity of the emitted light from *z* and with photon energy *E*. The final collected PL profile is based on the integration from *z*=0 to *z*=*L* of Eq. 1.

**Supplementary Note 3. Raman Spectral Analysis.**

Analysis of the low-frequency Raman spectra recorded from CsPbBr_3_ is based on the assumption that the quasi-elastic scattering background (i.e. zero-mode) is described by a Debye relaxation model and that it does not couple of interact with low-frequency modes of the perovskite. Therefore, we fit the Raman scattering intensity at different frequencies to the sum of a Debye relaxation and multiple damped Lorentz oscillators:

$I_{R}(\omega)=\frac{I(\omega)}{\omega[n(\omega)+1]}=C_{0}\frac{\gamma_{0}}{\omega^{2}+\gamma_{0}^{2}}+\sum_{j} C_{j}\frac{\omega_{j}^{2}\Gamma_{j}}{(\omega_{j}^{2}-\omega^{2})+\omega^{2}\Gamma_{j}^{2}}$ (2)

Here *I*(*ω*) is the experimental Raman scattering intensity and *n*(*ω*) + 1 is the Bose-Einstein population factor for the Stokes scattering. Parameters *ω*_j_ and *Γ*_j_ are respectively the frequency and damping of the *j*_th_ Lorentz oscillator and γ_j_ represents the inverse of the Debye relaxation time, through *τ*_r_ = 1/(2πcγ_0_), where *c* is the speed of light. The scattering constants of the Debye relaxation and the *j*_th_ phonon are represented by *C*_D_ and *D*_j_, respectively. For the temperature dependence of the zero-mode, as presented in the main text, we consider the integrated intensity^2^ and not factors *C*_D_ or *C*_j_.

Example of fits produced through this model are provided in Supplementary Figure S10, showing sound agreement with the experimental data at both high and low temperatures. The frequency range of the Raman spectra presented in the main article reflects the lower limit of the experimental detection window. As such, at higher temperatures, the spectral contributions arising from the Raman mode near 23 cm^-1^ are partially truncated, limiting the accuracy of a “blind” fitting procedure. To assist the fit of these high-temperature data, the RT parameters provided by Yaffe *et al.*^33^ (who evaluated the spectra down to lower wavenumbers) allows accurate limits on our fitting parameters. In this scenario, we find that we arrive at very nearly the same values for our RT fitting parameters, permitting the spectra measured at these temperatures to contribute reliably to our analysis. It is also within this context that further experimental ambiguity was introduced at some temperature change steps, where the absolute value of the scattering signal *i* shifted due to thermal compression and movement of the sample. To account for these, a renormalization of the spectra has been applied, as interpreted from by clear spectral trends.

**Supplementary Note 4. Fitting the monomolecular and bimolecular recombination coefficients *k*_1_ and *k*_2_ in perovskite single crystals.**

Since in SCs, the PL kinetics are sensitive to surface recombination, carrier diffusion, and reabsorption processes using one-phonon excitation, we chose two-photon absorption (800 nm excitation) to excite the single crystals. The PL dynamics can be modeled with an effective recombination upon two-photon excitation when the diffusion term is neglected:

$\begin{matrix} \frac{dn}{dt}=-f(T)B_{1}(T)np-(1-f(T))B_{2}(T)np-\frac{(1-f(T))n}{\tau} \\ \approx-f(T)B_{1}(T)n^{2}-\frac{(1-f(T))n}{\tau} \\ =-k_{2}(T)n^{2}-k_{1}(T)n \end{matrix}$ (3)

$n=\frac{k_{1}}{k_{2}}\frac{1}{\frac{k_{1}+k_{2}n_{0}}{k_{2}n_{0}}\exp(k_{1}t)-1}$ (4)

where *f*(*T*) is the ratio of carriers that undergoes radiative recombination at the direct band edge, *B*_1_(*T*) the e-h radiative recombination coefficient at the direct band edge, *B*_2_(*T*) is the e-h radiative recombination coefficient at the indirect band edge, which is small in SCs and can be omitted in the 1^st^ approximation. *τ* is the monomolecular recombination lifetime of the photoexcited carriers at the indirect band edge through mid-gap traps or with doped carriers. Auger recombination and exciton recombination are not considered in the model due to their limited contribution to low carrier density kinetics. *k*_1_ and *k*_2_ are the effective first-order and second-order recombination coefficients in the thermodynamic system, respectively.

The contribution from surface recombination is still observed, which, nonetheless, shows little carrier concentration dependence. The fast surface component is added to the fitting model as an independent exponential decay (rate: *k*_3_):

(5)

where *A*_1_ and *A*_2_ are surface and bulk weight ratio. Then three data sets with varied intensities are globally fitted using Eq. S4, from which *k*_1_, *k*_2_ are extracted. The average carrier concentration is estimated within a 50 μm thick top region as measured previously for MAPbBr_3_ SCs.^1^ The length corresponds to the penetration depth of the photon at Peak 2.

**Supplementary References**

1. Wu, B. *et al.* Discerning the Surface and Bulk Recombination Kinetics of Organic-Inorganic Halide Perovskite Single Crystals. *Adv. Energy Mater.* **6**, 1600551 (2016).

2. Chen, Y. *et al.* Extended carrier lifetimes and diffusion in hybrid perovskites revealed by Hall effect and photoconductivity measurements. *Nat. Commun.* **7**, 12253 (2016).

3. Wang, T. *et al.* Indirect to direct bandgap transition in methylammonium lead halide perovskite. *Energ. Environ. Sci.* **10**, 509-515 (2017).

4. Hutter, E. M. *et al.* Direct-indirect character of the bandgap in methylammonium lead iodide perovskite. *Nat. Mater.* **16**, 115-120 (2017).

5. Wehrenfennig, C., Liu, M. Z., Snaith, H. J., Johnston, M. B. & Herz, L. M. Charge-carrier dynamics in vapour-deposited films of the organolead halide perovskite CH3NH3PbI3-xClx. *Energ. Environ. Sci.* **7**, 2269-2275 (2014).

6. Rehman, W. *et al.* Charge-Carrier Dynamics and Mobilities in Formamidinium Lead Mixed-Halide Perovskites. *Adv. Mater.* **27**, 7938-7944 (2015).

7. Blancon, J. C. *et al.* The Effects of Electronic Impurities and Electron-Hole Recombination Dynamics on Large-Grain Organic-Inorganic Perovskite Photovoltaic Efficiencies. *Adv. Funct. Mater.* **26**, 4283-4292 (2016).

8. Trinh, M. T., Wu, X. X., Niesner, D. & Zhu, X. Y. Many-body interactions in photo-excited lead iodide perovskite. *J Mater. Chem. A* **3**, 9285-9290 (2015).

9. Milot, R. L., Eperon, G. E., Snaith, H. J., Johnston, M. B. & Herz, L. M. Temperature-Dependent Charge-Carrier Dynamics in CH3NH3PbI3 Perovskite Thin Films. *Adv. Funct. Mater.* **25**, 6218-6227 (2015).

10. Kandada, A. R. S. & Petrozza, A. Photophysics of Hybrid Lead Halide Perovskites: The Role of Microstructure. *Accounts Chem. Res.* **49**, 536-544 (2016).

11. Yang, Y. *et al.* Comparison of Recombination Dynamics in CH3NH3PbBr3 and CH3NH3PbI3 Perovskite Films: Influence of Exciton Binding Energy. *J. Phys. Chem. Lett.* **6**, 4688-4692 (2015).

12. Yamada, Y., Nakamura, T., Endo, M., Wakamiya, A. & Kanemitsu, Y. Photocarrier Recombination Dynamics in Perovskite CH3NH3PbI3 for Solar Cell Applications. *J. Am. Chem. Soc.* **136**, 11610-11613 (2014).

13. Manser, J. S. & Kamat, P. V. Band filling with free charge carriers in organometal halide perovskites. *Nat. Photon.* **8**, 737 (2014).

14. Wehrenfennig, C., Eperon, G. E., Johnston, M. B., Snaith, H. J. & Herz, L. M. High Charge Carrier Mobilities and Lifetimes in Organolead Trihalide Perovskites. *Adv. Mater.* **26**, 1584-1589 (2014).

15. Stranks, S. D. *et al.* Recombination Kinetics in Organic-Inorganic Perovskites: Excitons, Free Charge, and Subgap States. *Phys. Rev. Appl.* **2**, 034007 (2014).

16. Wehrenfennig, C., Liu, M. Z., Snaith, H. J., Johnston, M. B. & Herz, L. M. Charge carrier recombination channels in the low-temperature phase of organic-inorganic lead halide perovskite thin films. *Apl. Mater.* **2**, 081513 (2014).

17. Kong, W. G. *et al.* Characterization of an abnormal photoluminescence behavior upon crystal-phase transition of perovskite CH3NH3PbI3. *Phys. Chem. Chem. Phys.* **17**, 16405-16411 (2015).

18. Priante, D. *et al.* The recombination mechanisms leading to amplified spontaneous emission at the true-green wavelength in CH3NH3PbBr3 perovskites. *Appl. Phys. Lett.* **106**, 081902 (2015).

19. Fang, X. *et al.* Effect of excess PbBr2 on photoluminescence spectra of CH3NH3PbBr3 perovskite particles at room temperature. *Appl. Phys. Lett.* **108**, 071109 (2016).

20. Schmidt, T., Lischka, K. & Zulehner, W. Excitation-Power Dependence of the near-Band-Edge Photoluminescence of Semiconductors. *Phys. Rev. B* **45**, 8989-8994 (1992).

21. Murali, B. *et al.* Surface Restructuring of Hybrid Perovskite Crystals. *Acs Energy Lett.* **1**, 1119-1126 (2016).

22. Murali, B. *et al.* The Surface of Hybrid Perovskite Crystals: A Boon or Bane. *Acs Energy Lett.* **2**, 846-856 (2017).

23. Qiu, Y. H. *et al.* Tuning the Competitive Recombination of Free Carriers and Bound Excitons in Perovskite CH3NH3PbBr3 Single Crystal. *J. Phys. Chem. C* **121**, 6916-6923 (2017).

24. Miyata, A. *et al.* Direct measurement of the exciton binding energy and effective masses for charge carriers in organic-inorganic tri-halide perovskites. *Nat. Phys.* **11**, 582-U594 (2015).

25. Wright, A. D. *et al.* Electron-phonon coupling in hybrid lead halide perovskites. *Nat. Commun.* **7**, 11755 (2016).

26. Wakaiki, S. *et al.* Photoluminescence dynamics originating from exciton-exciton and exciton-electron scattering in a GaN thin film. *Phys. Status Solidi C* **9**, 2497-2500 (2012).

27. Kunugita, H. *et al.* Exciton-exciton scattering in perovskite CH3NH3PbBr3 single crystal. *Jpn. J. Appl. Phys.* **55**, 060304 (2016).

28. Klingshirn, C. F. *Semiconductor optics*. 3rd edn, Springer (2007).

29. Galkowski, K. *et al.* Determination of the exciton binding energy and effective masses for methylammonium and formamidinium lead tri-halide perovskite semiconductors. *Energ. Environ. Sci.* **9**, 962-970 (2016).

30. Wenger, B. *et al.* Consolidation of the optoelectronic properties of CH3NH3PbBr3 perovskite single crystals. *Nat. Commun.* **8**, 590 (2017).

31. Fang, Y. J., Wei, H. T., Dong, Q. F. & Huang, J. S. Quantification of re-absorption and re-emission processes to determine photon recycling efficiency in perovskite single crystals. *Nat. Commun.* **8**, 14417 (2017).

32. Roige, A. *et al.* Effects of photon reabsorption phenomena in confocal micro-photoluminescence measurements in crystalline silicon. *J. Appl. Phys.* **121**, 063101(2017).

33. Yaffe, O. *et al.* Local Polar Fluctuations in Lead Halide Perovskite Crystals. *Phys. Rev. Lett.* **118**, 136001(2017).
